# Supplementary material for: Superlattice by charged block copolymer self-assembly
Source: Nat Commun. 2019 May 8;10:2108. doi: 10.1038/s41467-019-10141-z (PMC6506472; doi:10.1038/s41467-019-10141-z)
Supplement: Supplementary file 1 — Supplementary Information [file 41467_2019_10141_MOESM1_ESM.pdf]

# Superlattice by charged block copolymer self-assembly

Shim et al.

## Supplementary Methods

**Materials.** Poly(ethylene glycol) methyl ether methacrylate ($M_n$ = 360 g mol$^{-1}$), poly(ethylene glycol) methacrylate ($M_n$ = 300 g mol$^{-1}$), and styrene were purchased from Aldrich and passed through a basic alumina column prior to polymerization. 2,2'–Azobis(isobutyronitrile) (AIBN, Aldrich) was recrystallized from methanol prior to use. The chain transfer agent, 2–cyano–2–propyl benzodithioate, was purchased from Aldrich and used as received. Sodium hydride (60% dispersion in mineral oil) and 1,3–propanesultone were purchased from Aldrich and stored in an argon-filled glove box. Ruthenium tetroxide ($RuO_4$) 0.5% aqueous solution was purchased from Electron Microscopy Sciences and used as received. All other chemicals and solvents were purchased from reliable commercial sources and used as received.

**Other characterization.** The number average molecular weights and molar compositions of the polymers were determined by $^1$H nuclear magnetic resonance (NMR) spectroscopy. All spectra were recorded on a Bruker Advance III HD (400 MHz) and DMSO–$d_6$ (Cambridge Isotope Laboratories) was used as a solvent. The dispersities ($Đ$) were determined by size exclusion chromatography (SEC) using dimethylformamide (DMF) containing 0.05 M lithium bromide (LiBr) as a mobile phase equipped with a laser-light scattering detector.

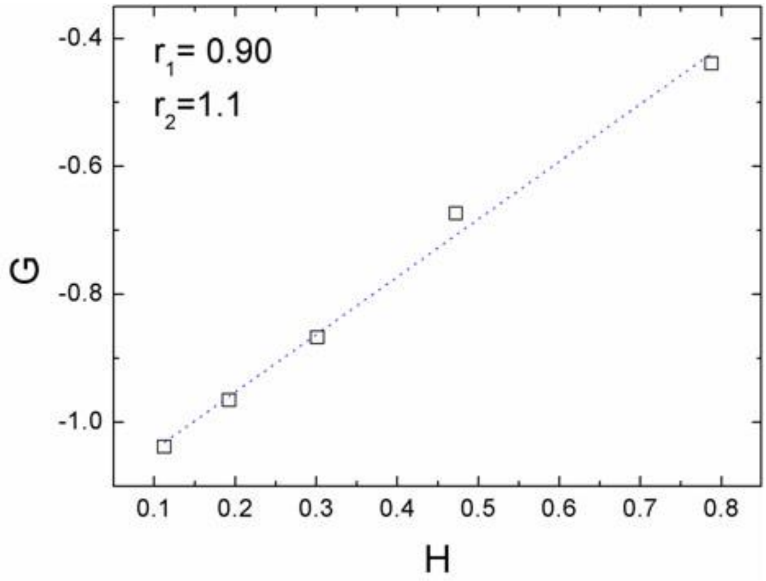


**Supplementary Fig. 1** Reactivity ratios. Determination of reactivity ratios of poly(ethylene glycol) methacrylate ($r_1$) and poly(ethylene glycol) methyl ether methacrylate ($r_2$) using Fineman–Ross method.

To confirm the random distribution of the two monomers in the POEGMA block, reactivity ratio of each monomer was determined by Fineman–Ross method which yielded quite comparable reactivity ratios, 0.90 and 1.1 for poly(ethylene glycol) methacrylate and poly(ethylene glycol) methyl ether methacrylate, respectively. This result postulates that both monomers are quite randomly distributed in the statistical copolymer system with less gradient or blocky feature since the product value of the reactivity ratios ($r_1r_2$) is nearly close to unity. The monomer reactivity ratios are obtained by the equation: $G = Hr_1 - r_2$ (1), where the reactivity ratios, $r_1$ and $r_2$ correspond to the reactivity ratio of poly(ethylene glycol) methacrylate and poly(ethylene glycol) methyl ether methacrylate, respectively. The parameters G and H are defined as $G = X(Y - 1)$ (2) and $H = X^2/Y$ (3), respectively, where $X = M_1/M_2$ (4) and $Y = dM_1/dM_2$ (5), in which $M_1$ and $M_2$ are the monomer molar compositions in the feed and $dM_1$ and $dM_2$ are the compositions in the copolymer. Note that the polymerization was conducted below 10% of conversion for the determination of the reactivity ratios.

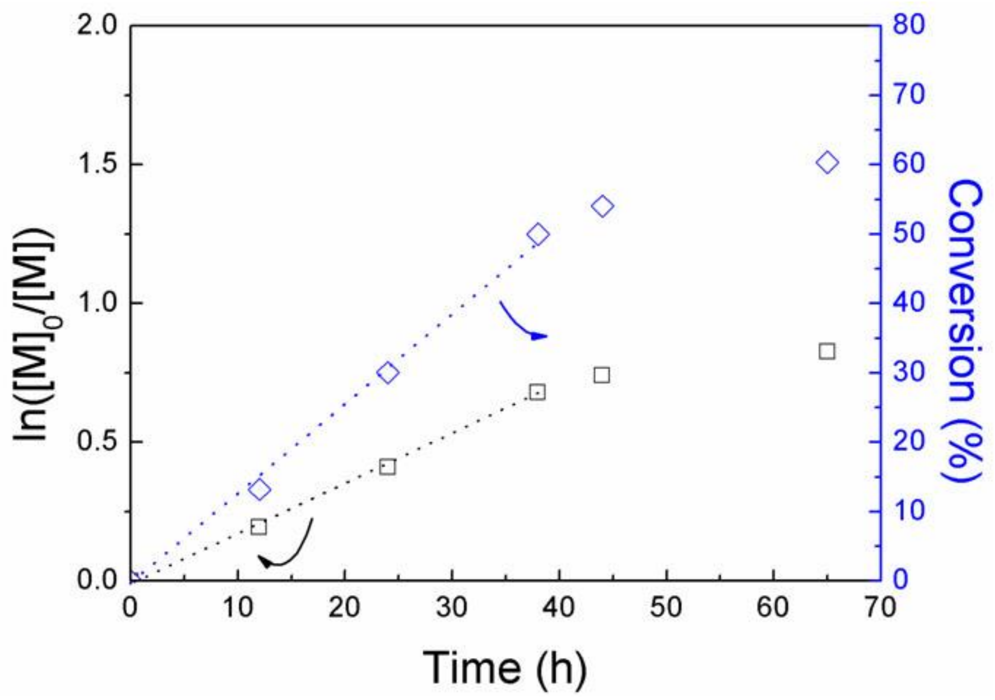


**Supplementary Fig. 2** Living characteristics of RAFT polymerization. First-order kinetic plot of POEGMA23–PS as a function of reaction time.

Supplementary Fig. 2 shows the characteristic linear kinetic plot of POEGMA23–PS which shows the evolution of the molecular weight and conversion with the reaction time, where slight curvature appears at the extended polymerization time probably due to the termination at the prolonged reaction time. Thus, polymerization was conducted until the styrene conversion reaches 50% (corresponding reaction time: 38 h) which falls within the first-order kinetic regime. All the samples in the series of POEGMA–PS also exhibit nearly same linear kinetic with POEGMA23–PS.

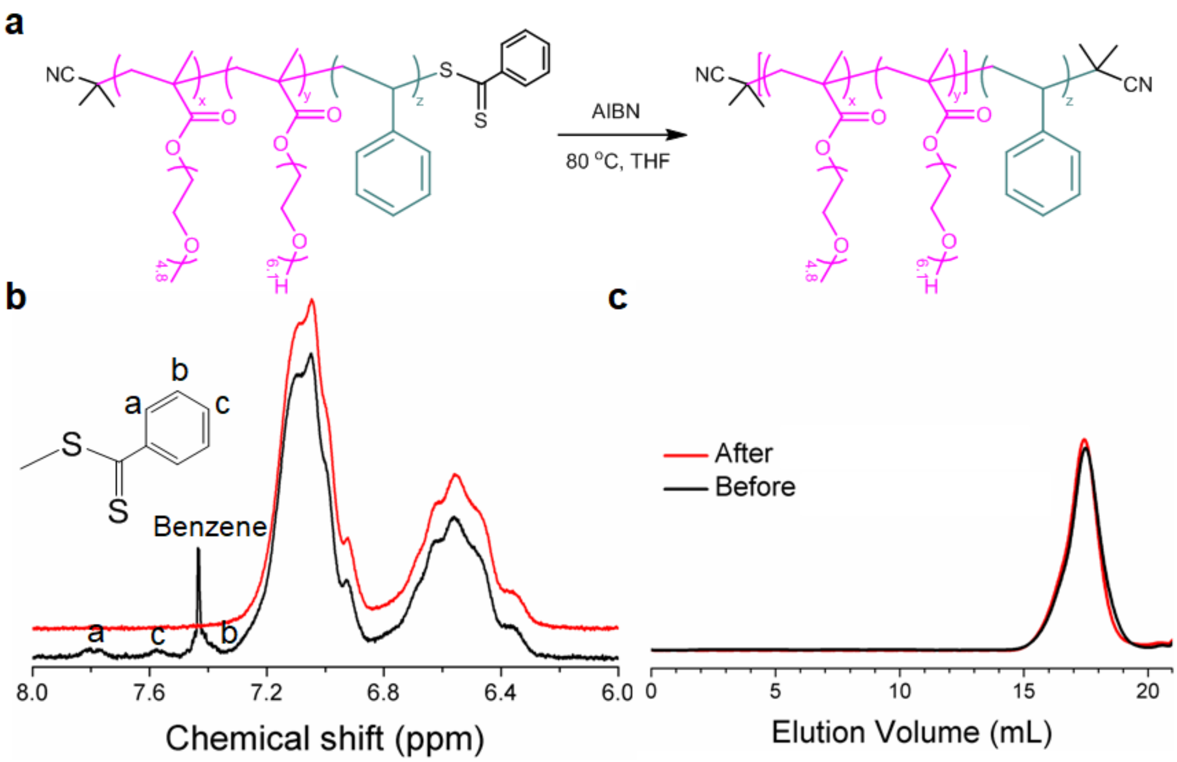


**Supplementary Fig. 3** Synthesis and characterization of POEGMA23–PS. **a** End group substitution by radical-induced cross-coupling reaction. **b** Representative $^1$H NMR spectra and **c** SEC trace of POEGMA23–PS before and after the end group substitution.

Prior to introducing the ionic species to the hydroxyl groups in the POEGMA block, end group originating from the RAFT chain transfer agent was substituted from the reactive dithioester group to the inert isopropyl cyanide group by radical-induced cross-coupling reaction as presented in Supplementary Fig. 3a to preclude the possible side reactions during the post-modification. As shown in Supplementary Fig. 3b, the end group proton signals, a, b, and c, completely disappear from the $^1$H NMR spectra after the reaction, while the SEC traces before and after the chemical substitution are almost identical as presented in Supplementary Fig. 3c.

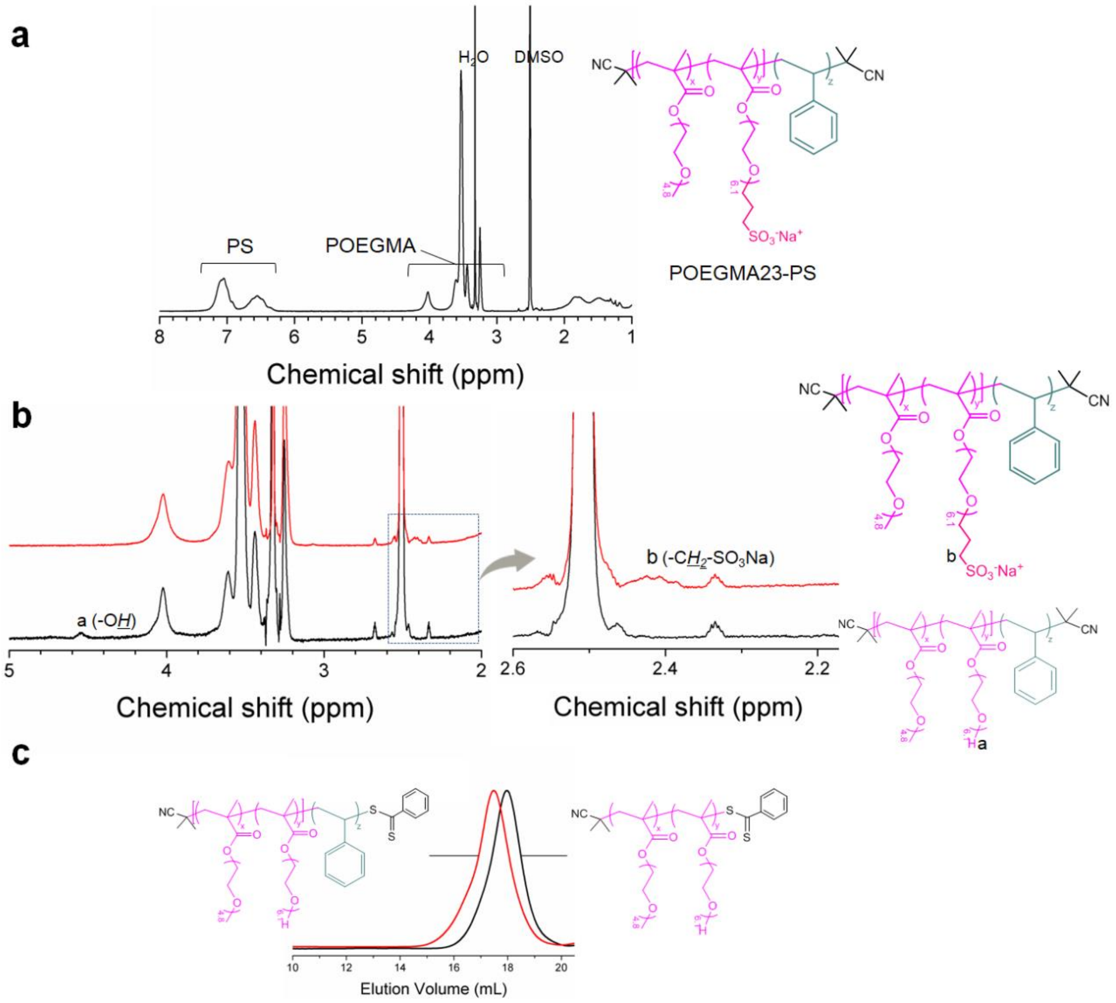


**Supplementary Fig. 4** Chemical structure confirmation. Representative $^1$H NMR spectra of **a** POEGMA23–PS and **b** before and after introduction of sodium sulfonate groups. **c** Representative SEC traces of macro chain transfer agent and the resulting block copolymer.

All the hydroxyl groups in the POEGMA block were modified to sodium sulfonate groups by the reaction with 1,3–propanesultone. Supplementary Fig. 4a shows $^1$H NMR spectrum of the resulting POEGMA23–PS. The conversion of the post-modification reaction was 100% which was confirmed by $^1$H NMR spectroscopy as shown in Supplementary Fig. 4b. Upon the post-modification through the hydroxyl groups, hydroxyl proton peaks at 4.55 ppm (signal a) clearly disappears, while the new –C$H_2$–$SO_3$Na peaks at 2.33 ppm (signal b) are generated. Supplementary Fig. 4c shows the SEC traces of macro chain transfer agent and the resulting block copolymer.

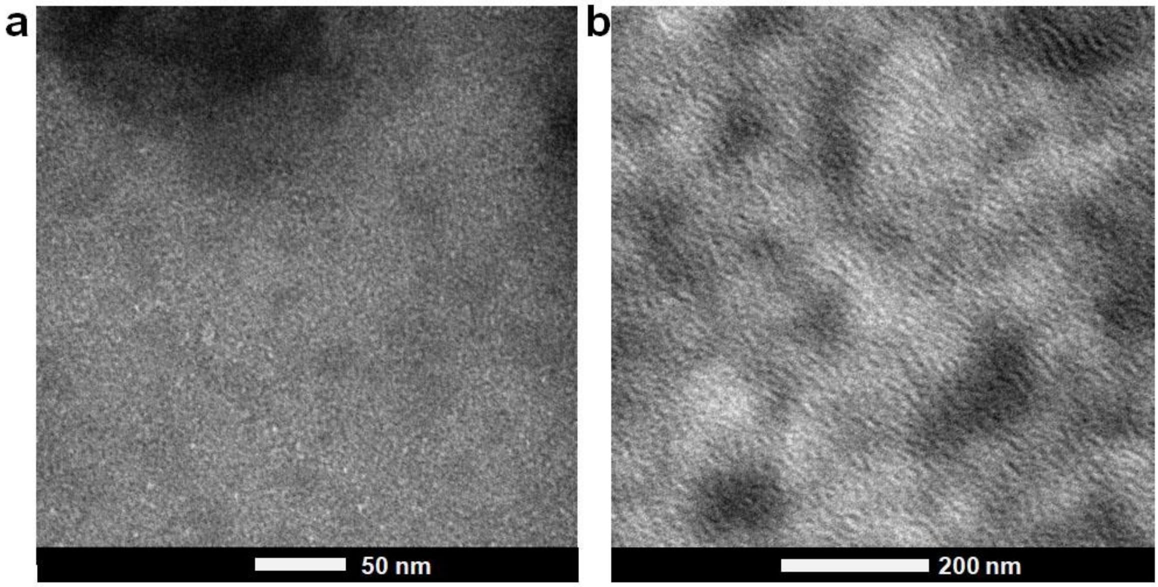


**Supplementary Fig. 5** Real-space images. TEM image of **a** POEGMA0–PS and **b** POEGMA36–PS.

As shown by Supplementary Fig. 5a, TEM image of POEGMA0–PS shows completely disordered phase, whereas the low-magnification TEM image of POEGMA36–PS in Supplementary Fig. 5b displays a lamellar structure.

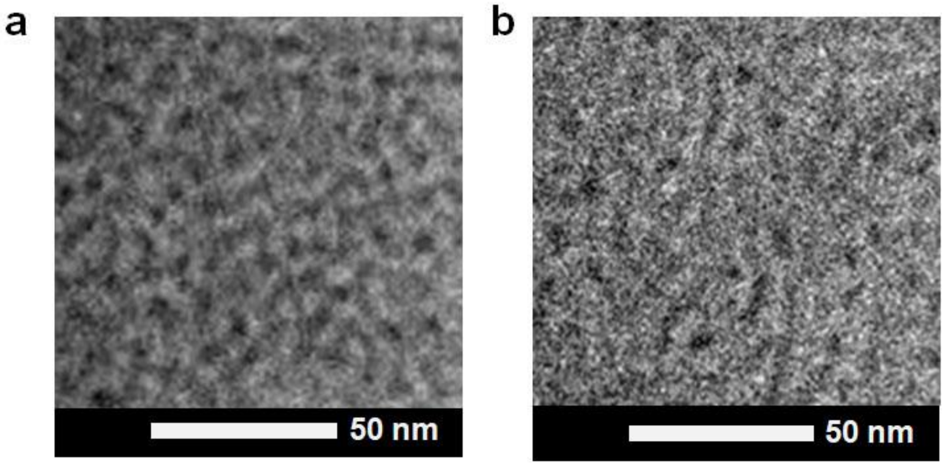

**Supplementary Fig. 6** Real-space images. TEM images of **a** POEGMA7–PS and **b** POEGMA23–PS.

Since the hygroscopic nature of POEGMA7–PS and POEGMA23–PS significantly affects the morphological disruption during the staining process using 0.5% $RuO_4$ aqueous solution, the morphologies shown by the TEM images in Supplementary Fig. 6 are not consistent with the SAXS data in Fig. 2, in that no long-range order was observed in the TEM images.

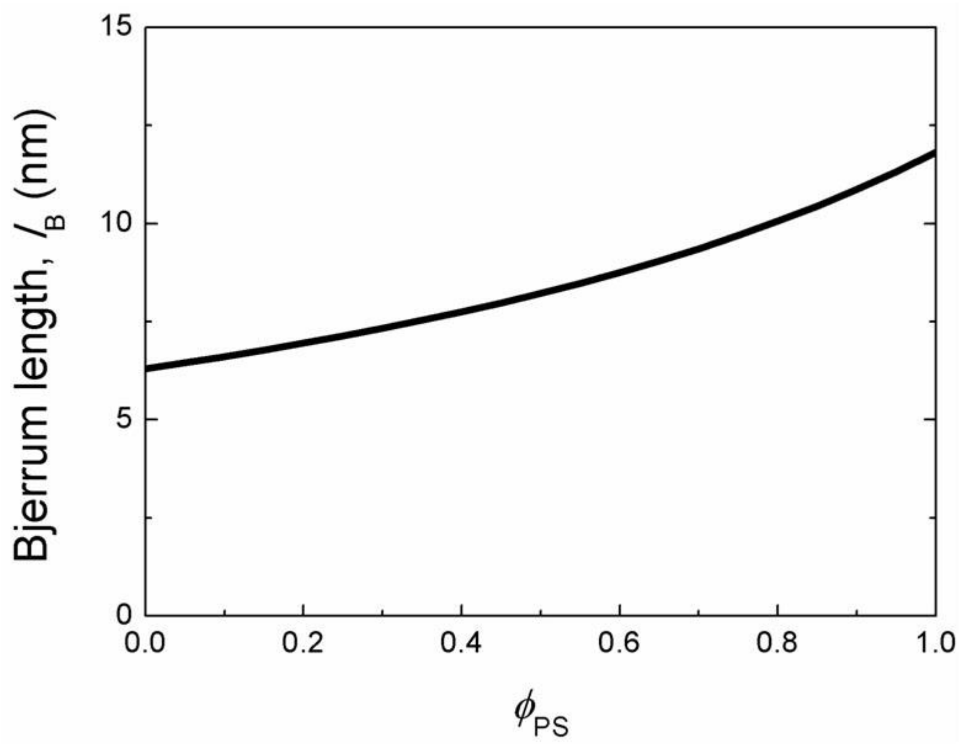


**Supplementary Fig. 7** Electrostatic correlations. Bjerrum length ($l_B$) as a function of local volume fraction of PS ($\phi_{PS}$) (temperature: 80 °C).

The Bjerrum length ($l_B$), which reflects the electrostatic cohesion strength, was estimated. Supplementary Fig. 7 shows the $l_B$ values as a function of local volume fraction of PS ($\phi_{PS}$). The $l_B$ values of our system is in the range of 6–11 nm depending on the local volume fraction of each POEGMA and PS phase, while the real value should be closer to 6 nm since the ionic species tend to be preferentially solubilized in POEGMA phase. Meanwhile, the average spacing between the charged groups by assuming they are all in the POEGMA domains and distributed uniformly, in POEGMA7–PS and POEGMA23–PS is 2.2 nm and 1.2 nm, respectively.

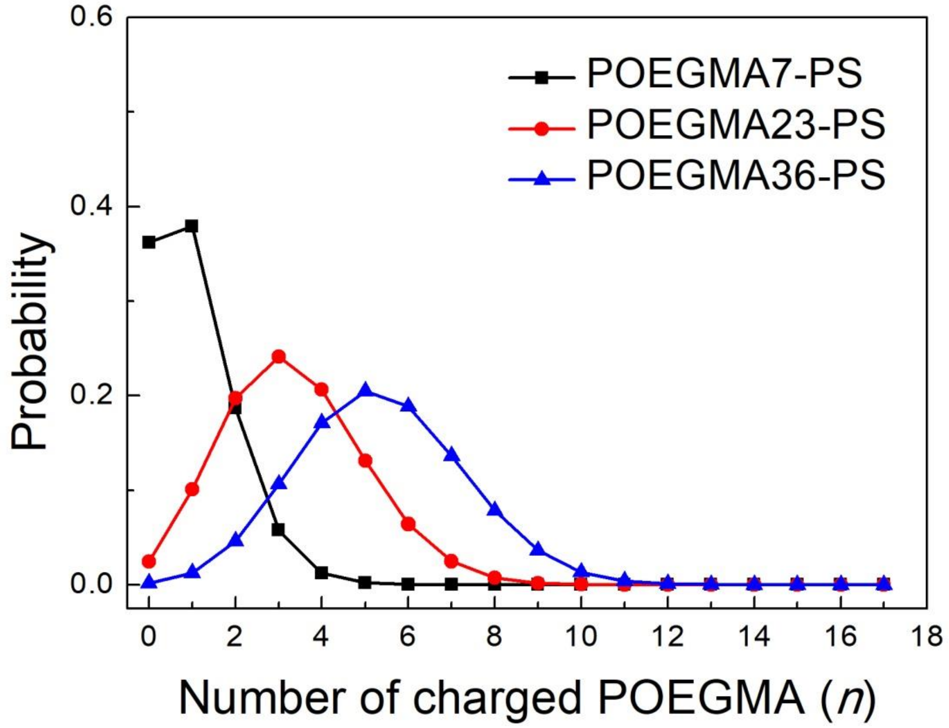


**Supplementary Fig. 8** Dispersity in the number of charges per chain. Composition distribution probability profiles of POEGMA#–PS (#: 7, 23, and 36).

The component distribution probability profiles of POEGMA#–PS system can be estimated by assuming a simple binomial distribution of charged and uncharged POEGMA monomers along the backbone. The probability that the number of charged POEGMA monomers in POEGMA block equals $n$ is

$$[17!/\{n!(17-n)!\}]\times[\{r_{neutral}/(r_{neutral}+r_{charged})\}\times f_{neutral}]^{17-n}\times[\{r_{charged}/(r_{neutral}+r_{charged})\}\times f_{charged}]^{n}$$

where $r_{neutral}$ (=1.1) and, $r_{charged}$ (=0.9) are the reactivity ratios of neutral and charged POEGMA monomer, respectively, and each set of ($f_{neutral}$, $f_{charged}$) = (0.93, 0.07), (0.77, 0.23), and (0.64, 0.36) is the molar feed ratio of neutral and charged POEGMA monomer for synthesizing POEGMA7–PS, POEGMA23–PS, and POEGMA36–PS, respectively. To simplify the calculation, the degree of polymerization of each POEGMA block was assumed to be 17.

As shown in Supplementary Fig. 8, POEGMA7–PS, POEGMA23–PS, and POEGMA36–PS exhibit a probability of possessing uncharged diblock copolymer ($n$ = 0) of 0.36, 0.024, and 0.0016, respectively. Since POEGMA7–PS has only a few charged species along the backbone with a relatively small degree of polymerization of 17, it has a greater probability of possessing a substantial amount of uncharged block copolymers than POEGMA23–PS and POEGMA36–PS. Thus, as the targeting charge fraction decreases, it is more likely to be a mixture of uncharged and charged block copolymers.
